# CDAE: A Cascade of Denoising Autoencoders for Noise Reduction in the Clustering of Single-Particle Cryo-EM Images

**DOI:** 10.3389/fgene.2020.627746

**Published:** 2021-01-20

**Authors:** Houchao Lei, Yang Yang

**Affiliations:** ^1^Center for Brain-Like Computing and Machine Intelligence, Department of Computer Science and Engineering, Shanghai Jiao Tong University, Shanghai, China; ^2^Key Laboratory of Shanghai Education Commission for Intelligent Interaction and Cognitive Engineering, Shanghai, China

**Keywords:** cryo-EM, autoencoder, image denoising, clustering, deep learning

## Abstract

As an emerging technology, cryo-electron microscopy (cryo-EM) has attracted more and more research interests from both structural biology and computer science, because many challenging computational tasks are involved in the processing of cryo-EM images. An important image processing step is to cluster the 2D cryo-EM images according to their projection angles, then the cluster mean images are used for the subsequent 3D reconstruction. However, cryo-EM images are quite noisy and denoising them is not easy, because the noise is a complicated mixture from samples and hardware. In this study, we design an effective cryo-EM image denoising model, CDAE, i.e., a cascade of denoising autoencoders. The new model comprises stacked blocks of deep neural networks to reduce noise in a progressive manner. Each block contains a convolutional autoencoder, pre-trained by simulated data of different SNRs and fine-tuned by target data set. We assess this new model on three simulated test sets and a real data set. CDAE achieves very competitive PSNR (peak signal-to-noise ratio) in the comparison of the state-of-the-art image denoising methods. Moreover, the denoised images have significantly enhanced clustering results compared to original image features or high-level abstraction features obtained by other deep neural networks. Both quantitative and visualized results demonstrate the good performance of CDAE for the noise reduction in clustering single-particle cryo-EM images.

## 1. Introduction

Recent progress of cryo-electron microscopy (cryo-EM) has revolutionized the field of structural biology (Cheng et al., 2015). Thanks to this technology, more and more spatial structures of bio-molecules with nearly atomic-resolution have been solved. In order to obtain the 3D structure of a macromolecular, a large amount of 2D projection images with various orientations are captured, processed and averaged for reconstruction. At present, there are some softwares to realize the whole 3D reconstruction process, such as SPREAD (Xie et al., 2020). The whole pipeline involves quite a few scientific problems with great challenges in computation and algorithms.

During the preprocessing steps of images before 3D reconstruction, there are some major computational tasks listed in the following:

1. Estimation of the contrast transfer function (CTF) induced by the underfocus issue (Penczek et al., 1997). Specialized image processing algorithms such as phase flipping and amplitude correction/wiener filtering can or partially correct the CTF (Downing and Glaeser, 2008);
2. Automatic particle picking, i.e., recognizing and extraction of the particles from micrographs. Some popular software packages, like XMIPP (de la Rosa-Trevín et al., 2013), provide GUI programs to help pick projection images semi-automatically;
3. Clustering images by their projection angles. The images within clusters are averaged for 3D reconstruction, In addition to the common clustering methods such as kmeans, IterVM (Ji et al., 2018) proposes an iterative clustering model based on convolutional autoencoder model to solve the single particle clustering problem in cryo electron microscopy;
4. Identification of structural heterogeneity. The raw images often exhibit different conformations due to various reasons. In order to obtain high-resolution structures, different conformations should be distinguished and classified into homogeneous groups.

Solving the last two tasks largely relies on unsupervised learning algorithms, since in the real cryo-EM images, each particle's orientation is random and unknown, and the conformation information is also absent. The clustering result has a substantial impact on the sub-sequent reconstruction quality, as the projection images with dissimilar angles will dramatically decrease the qualities of class average images, which are the reconstruction inputs. Due to the low electron dose limitation (to prevent radiation damage), the cryo-EM images usually have too much noise, leading to extremely low signal-to-noise ratios (SNRs), which greatly increases the complexity of particle picking and clustering of images. However, the existing clustering algorithms are general-purpose methods, few of them are designed for such low-SNR scenario. Besides, denoising is not easy for cryo-EM images because the noise is a complicated mixture from samples and hardware. Therefore, how to reduce noise and improve the clustering performance has become a crucial problem for the structure reconstruction.

In this paper, we focus on noise reduction for the clustering of cryo-EM images. Especially, we design an image denoising model, CDAE, which is a cascade of denoising autoencoders to reduce noise in a progressive manner. The model comprises 3 blocks, each of which is pre-trained by a simulated data set and fine-tuned by the target data set. We evaluate the performance of the new model on both simulated and real data sets. The results show that CDAE achieves much higher PSNR (peak signal-to-noise ratio) than the state-of-the-art denoising methods, and it significantly improves the performance of conventional clustering methods compared with the clustering based on original images or feature representations yielded by other models.

To summarize, the contributions of this study are two folds:

1. In order to deal with the extremely low signal-to-ratio in cryo-EM images, we propose a cascade architecture, which consists of a stack of autoencoders, for denoising in a progressive manner.
2. In order to address the unsupervised denoising problem, we propose a two-phase learning strategy, including pre-training using simulated data and fine-tuning using real data. The strategy improves the denoising performance of autoencoders effectively.

## 2. Related Work

### 2.1. Autoencoders for Feature Learning and Denoising

Autoencoder is a kind of unsupervised neural network, which comprises two parts, namely encoder and decoder. Encoder defines a parameterized function to extract features while decoder attempts to reconstruct original data from encoded features. The basic idea is to extract features through minimizing the reconstruction error.

Till now, various variants have been proposed to regularize the model. For instance, sparse autoencoder imposes a sparsity penalty on the latent layer to enforce sparsity of the features (Lee et al., 2007; Scholkopf et al., 2007). Instead of adding a penalty to the cost function, denoising autoencoder (DAE) (Vincent et al., 2008) attempts to reconstruct the original data from corrupted ones, which promotes the model to learn more useful and robust features. Following the DAE, contractive autoencoder (CAE) (Rifai et al., 2011) adds an analytic contractive penalty, which is a generalization of DAE. More recently, variational autoencoder (VAE)(Kingma and Welling, 2014) and adversarial autoencoders (AAE) (Makhzani et al., 2015) were designed to constraint the distribution of hidden variables. Most of these models aim to provide latent feature representations (dimensionality reduction) for subsequent learning, and some of them have been directly used for unsupervised clustering. For instance, GMVAE (Dilokthanakul et al., 2016) models the latent feature distribution as a Gaussian mixture distribution to cluster the latent vectors, and AAE could also serve as a clustering method when modeling the latent variables as a categorical distribution (Makhzani et al., 2015).

Besides, autoencoders have also been introduced in the denoising tasks. LeCun and Gallinari (Gallinari et al., 1987; Le Cun, 1987) pioneered the studies using autoencoders for noise reduction, and (Memisevic, 2007) designed a gated autoencoders for denoising. Note that denoising autoencoder (DAE) (Vincent et al., 2008) gets the name because its inputs are corrupted data, while its training objective is obtaining robust features rather than denoising.

### 2.2. Clustering of Cryo-EM Images

In recent years, various software packages for cryo-EM image processing have been released, many of which contain the clustering function. Some of them use *k*-means based clustering algorithm, such as XMIPP (Scheres et al., 2008). The clustering module of XMIPP is an implementation of CL2D algorithm (Sorzano et al., 2010), which is a modified *K*-means method. CL2D uses cross-entropy as the measurement of image similarity and proposes a new clustering criterion to address the varied SNR issue. Another well-known package, Spider (Frank et al., 1996), implements hierarchical clustering. These methods perform distance calculation directly using raw images.

Besides the conventional clustering methods, new algorithms specialized for cryo-EM images have also emerged. Relion (Scheres, 2012) developed a maximum likelihood (ML) based approach, aiming to find the optimal probability estimation, which is more robust to the influence of noise than traditional methods, but it is incompetent in differentiating subtle structural heterogeneity. Recently, a new software package ROME (Wu et al., 2016) was proposed, which introduces a new kind of clustering method based on statistical manifold learning (SML). The basic idea is to map the original data space into a lower dimensional latent space by a non-linear transformation, and then optimize the parameters by expectation-maximization (EM) algorithm.

## 3. Methodology

### 3.1. Problem Description

In a basic autoencoder model, the input and target output are the same; while our goal is noise reduction, thus the input and target output in our model are different. Let *X* and *Y* denote the sets of original noisy images and target clean images, respectively. We want to find a mapping function f:X↦Y, as formulated in Equation (3),

(1) $$\begin{array}{l} z=EC\left(x\right), \end{array}$$

(2) $$\begin{array}{l} y=DC\left(z\right), \end{array}$$

(3) $$\begin{array}{l} y=f\left(x\right)=DC\left(EC\left(x\right)\right), \end{array}$$

where *x* ∈ *X*, *y* ∈ *Y*, and *z* is the latent representation. *EC* is an encoder, and *DC* is a decoder.

**Figure 1:**
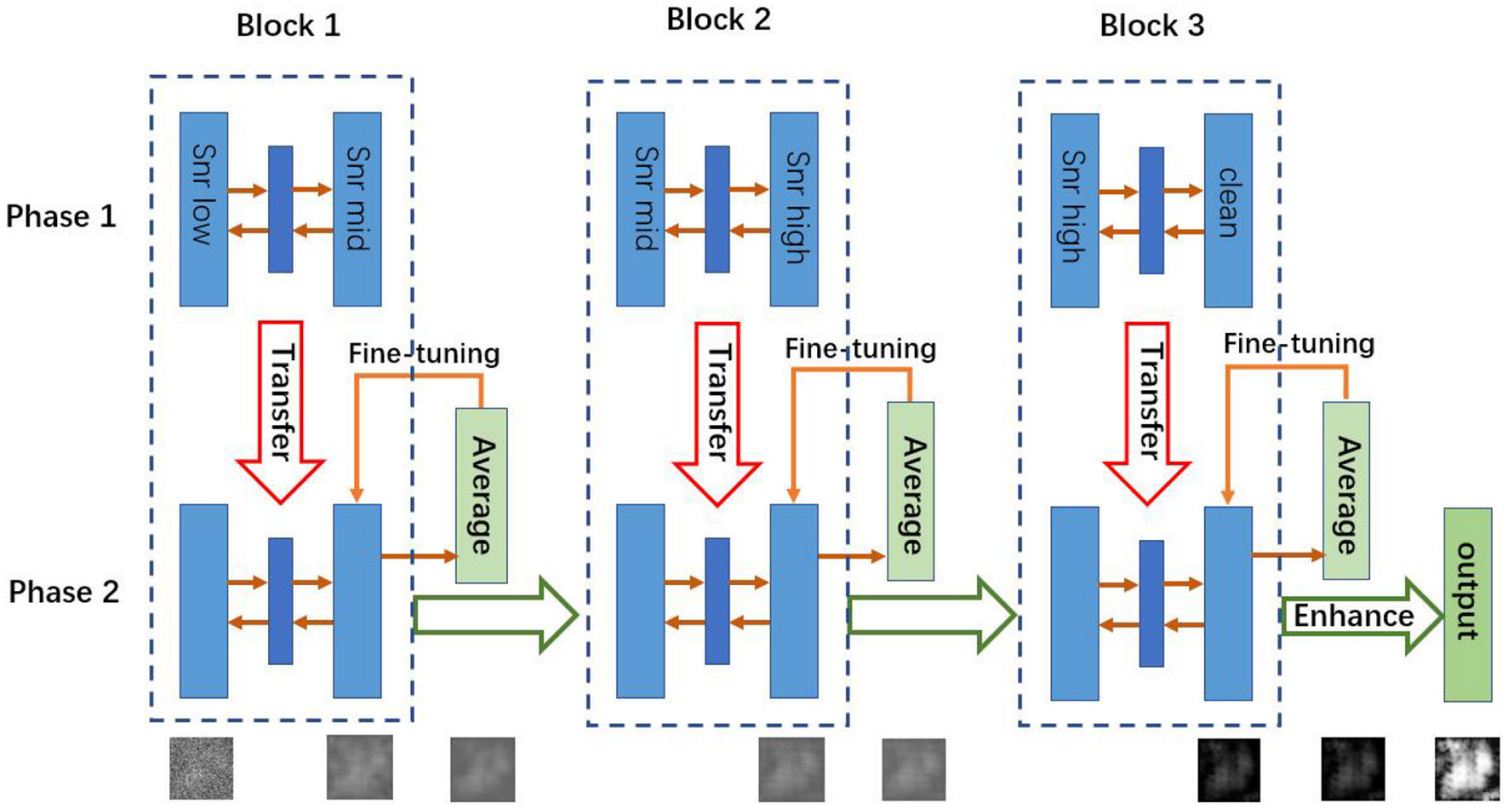
Architecture of CDAE. The algorithm has two phases. In Phase 1, for each block, the autoencoder is pre-trained by using the training data. Then the learned weights are transferred into Phase 2, where the autoencoders are fine-tuned by test data sets. Note that in Phase 1, the three blocks can be trained parallely, while in Phase 2, they are fine-tuned sequentially.

In a supervised learning scenario, the mapping function *f* can be learned from training data, but our task is unsupervised, because real cryo-EM images have no clean targets. In order to address this problem, we convert the original task into a supervised learning problem and adopt a two-phase learning strategy as shown in Figure 1. First, we pre-train the autoencoders with simulated paired cryo-EM data, which has the clean target image for training, and then we fine-tune the model with real data to transfer knowledge from simulated cryo-EM data to real data. These two phases are described in sections 3.2, 3.3, respectively.

### 3.2. Pre-training

Let *X*_*tr*_ and *Y*_*tr*_ denote the sets of the corrupted images and target images of the simulated training data, respectively. And $x_{tr}^{\left(i\right)}\in X_{tr}$ is an input image for the encoder, where *i* ∈ {1, 2, …, *n*} and *n* is the number of training images. The parameters, θ = {*W, b*} for *EC* and ϕ = {*W*′, *b*′} for *DC*, are optimized to minimize the average reconstruction error as shown in Equation (4),

(4) $$\begin{array}{l} \theta^{*},\phi^{*}=\underset{\theta ,\phi }{\arg\min}\frac{1}{n}\overset{n}{\underset{i=1}{\sum }}L\left(y_{tr}^{\left(i\right)},DC_{\phi }\left(EC_{\theta }\left(x_{tr}^{\left(i\right)}\right)\right)\right), \end{array}$$

where *L* is the loss function, such as mean-square-error.

### 3.3. Fine-Tuning

Let *X*_*te*_ denote the sets of the images of test dataset, i.e., real data, and $x_{te}^{\left(i\right)}\in X_{te}$, where *i* ∈ {1, 2, …, *m*} and *m* is the number of test images. *DC*′ and *EC*′ are pre-trained decoder and encoder, respectively. The parameters, θ′ of *EC*′ and ϕ′ of *DC*′, are further optimized to minimize the average reconstruction error as shown in Equation (5),

(5) $$\theta^{\prime }*,\phi^{\prime }*=\underset{\theta^{\prime },\phi^{\prime }}{\arg\min}\frac{1}{m}\sum_{i=1}^{m}L(\bar{x_{te}^{\left(i\right)}{\;}^{\prime }},D{C^{\prime }}_{\phi^{\prime }}(E{C^{\prime }}_{\theta^{\prime }}(x_{te}^{\left(i\right)}))),$$

(6) $$\begin{array}{l} {x_{te}^{\left(i\right)}}^{\prime }=DC_{\phi^{\prime }}^{\prime }\left(EC_{\theta^{\prime }}^{\prime }\left(x_{te}^{\left(i\right)}\right)\right), \end{array}$$

where ${x_{te}^{\left(i\right)}}^{\prime }$ is the corresponding output of $x_{te}^{\left(i\right)}$ by using *EC*′ and *DC*′ (Equation 6), and $\bar{{x_{te}^{\left(i\right)}}^{\prime }}$ is the mean image of ${x_{te}^{\left(i\right)}}^{\prime }$ averaged over its neighborhood, which is determined by a certain similarity metric and a threshold. Since there is no known clean data for test data, the mean images are used as target output instead. We use mean images as the targets because images of close orientations or conformations have similar features, but the noises mostly due to random events are not similar in these images. Thus the mean images will weaken the influence of noise and it could be regarded as a substitute for the target images without noise.

It is worth noting that we use the same data set in the fine-tuning stage and the test stage. However, in the fine-tuning stage, we only use the images of the test dataset, but not the targets of the test dataset. We use the mean images averaged over each image's neighbors as the target for training; while in the test stage, we use images and targets of test dataset to calculate the corresponding quantitative metrics.

### 3.4. The Cascade Design

The proposed CDAE model is a cascade of denoising autoencoders, which aims to reduce noise in a progressive manner for the images with very low SNR. As shown in Figure 1, CDAE has three blocks, each of which contains a convolutional autoencoder. During the pre-training phase, the first block learns the mapping from the images with a low SNR (SNR_*low*_) to images with a medium SNR (SNR_*mid*_), the second block learns from data of SNR_*mid*_ to data of SNR_*high*_, and the last layer learns from data of SNR_*high*_ to clean data. Then, we fine-tune the blocks sequentially from Block 1 to Block 3. The outputs of the fine-tuned blocks are fed to the next block. Finally, we make a histogram equalization enhancement to the output images of the last block. The procedure is described in Algorithm 1.

**Algorithm 1:** The CDAE Algorithm

**Input:** The training data sets: *X*_*tr,i*_(*i* ∈ {1, 2, 3, 4})^*a*^, and the test data set *X*_*te*_;
**Output:** Denoised image $X_{te}^{*}$;
1: Train the three blocks separately and obtain the mapping function *f*_*j*_ from *X*_*tr,j*_ to *X*_*tr,j*+1_, i.e., *f*_*j*_:*X*_*tr,j*_ ↦ *X*_*tr,j*+1_, *j* ∈ {1, 2, 3}
2: $X_{te}^{1}=X_{te}$
3: Fine-tune Block 1 and obtain the updated mapping function $f_{1}^{\prime }$, i.e., $f_{1}^{\prime }:X_{te}\mapsto \bar{f\left(X_{te}^{1}\right)}$
4: **for** *j* ∈ {2, 3} **do**
5: $X_{te}^{j}=f_{j-1}^{\prime }\left(X_{te}^{j-1}\right)$
6: $f_{1}^{\prime }:X_{te}^{j}\mapsto \bar{f\left(X_{te}^{1}\right)}$ ($f_{j}^{\prime }$ is initialized by *f*_j_)
7: **end for**
8: $X_{te}^{*}=Enhance\left({f_{3}}^{\prime }\left(X_{te}^{3}\right)\right)$
9: **return** $X_{te}^{*}$;
^*a*^ *X*_*tr*,1_, *X*_*tr*,2_, *X*_*tr*,3_ and *X*_*tr*,4_ denote the training sets with SNR_*low*_, SNR_*mid*_, SNR_*high*_ and no noise, respectively.

### 3.5. Architecture of the Model Components

**Figure 2:**
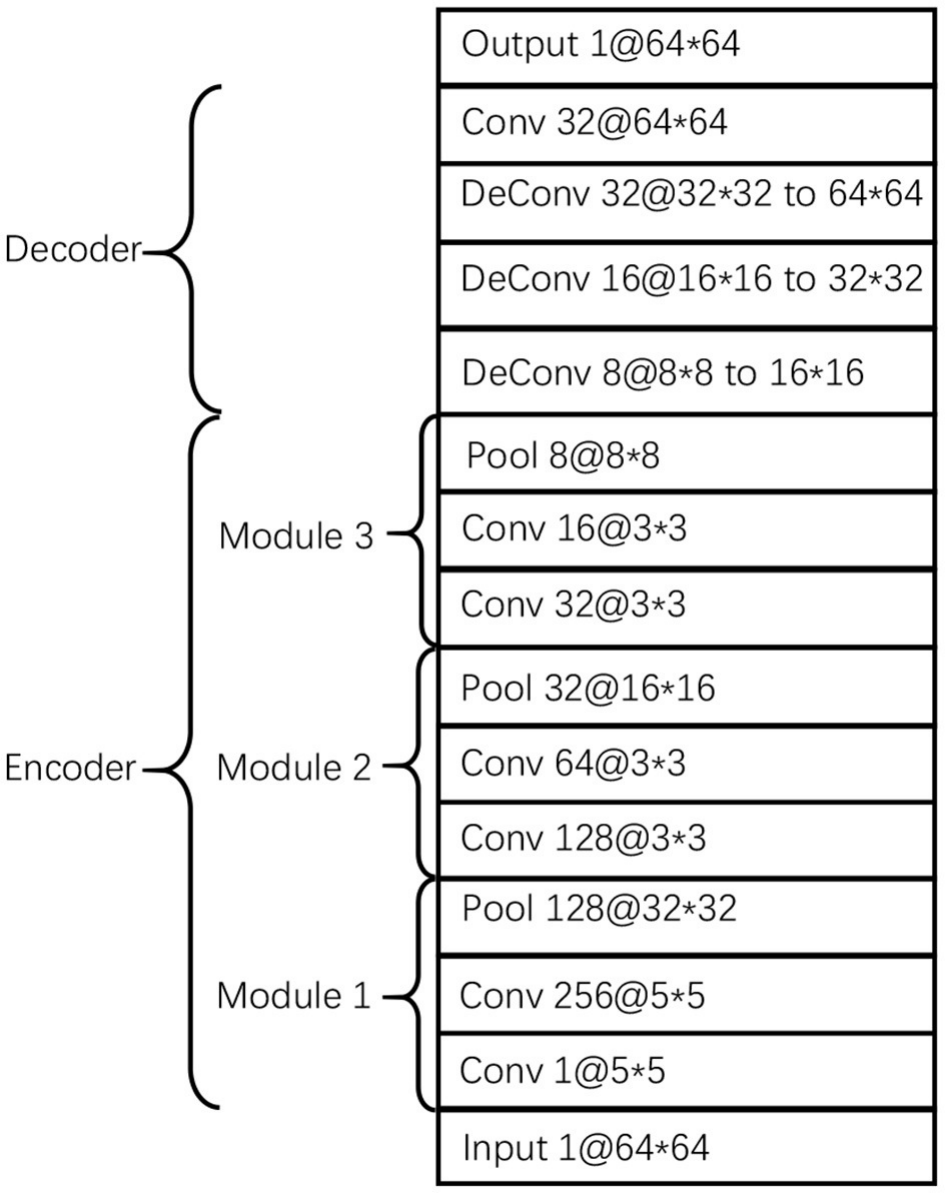
Parameters of the autoencoders in the three blocks of CDAE.

The proposed CDAE model comprises three components/blocks. Considering the advantages of convolutional neural networks in representing image features, we build a convolutional autoencoder in each block. The three autoencoders use the same parameters as listed in Figure 2. The encoder consists of 3 modules, each of which contains 2 convolutional layers and a pooling layer; while the decoder consists of 4 layers, including 3 deconvolutional layers and a convolutional layer. The function of the last convolution layer is to combine 32 channels into one channel as output. In order to avoid overfitting, we use dropout in the encoder and decoder and set dropout rate to 0.5.

## 4. Experimental Results

### 4.1. Dataset and Experimental Setup

**Figure 3:**
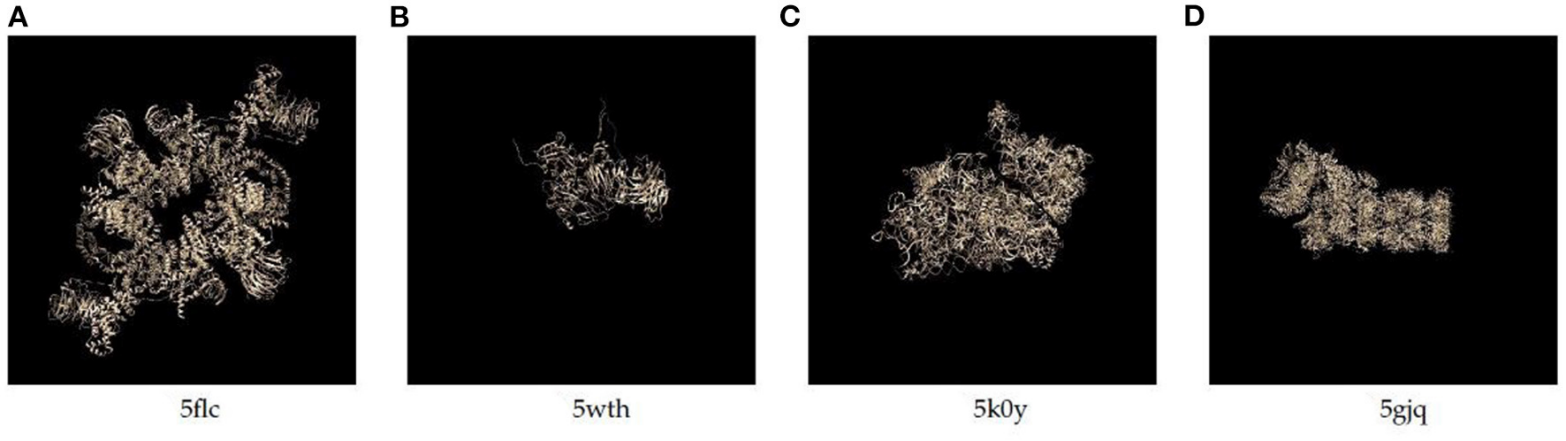
Structures of the 4 proteins used in our data sets.

We collect molecular structure data from the Electron Microscopy Data Bank (EMDB) at PDBe (Sameer et al., 2016),and prepare two kinds of data, including the data simulated by ourselves and real data downloaded from EMDB. For the simulated data, we extract the 3D structures of 4 proteins from the EMDB database, whose PDB IDs are 5wth, 5k0y, 5flc, and 5gjq, and their real structures are present in Figure 3. We simulate their 2D EM projection images by using the cryo-EM data processing suitcase software, XMIPP (de la Rosa-Trevín et al., 2013), which has been widely used in cryo-EM data processing and protein reconstruction task. In our experiment, we take the 2D images of 5flc as the training data (for pre-training the model), and images of the other 3 proteins as the test data. For 5flc, we simulate images with 4 different noise ratios (SNR_*low*_, SNR_*mid*_, SNR_*high*_ and no noise) and 4 orientations. The number of images with the same orientation and SNR is 1,000. Thus, there is a total of 4 × 4 × 1, 000 = 16, 000 pictures; while for the other 3 proteins, we only simulate the images with SNR_*low*_ at four orientations, thus each of which has 4, 000 pictures. In addition, the SNR_*low*_, SNR_*mid*_ and SNR_*high*_ used for simulation are set to 0.1, 0.4, 0.6, respectively. And, the number of closest neighbors (*k*) for obtaining mean images is set to 30.

Beside the simulated data, we also retrieve a real data set from EMDB, the cryo-EM images of GroEL (PDB entry 10029), where the simulation condition is 300 kV acceleration voltage. Since there is no orientation or conformation information in the data set, here we only show the visualized results (see section 4.5), i.e., the mean images from the clusters of denoised images.

### 4.2. Evaluation Criteria

In order to assess the new model, we provide both quantitive results (denoising and clustering experiments) and visualized results. The measurement of denoising performance lies in the similarity between reconstruction data and the clean data, while the clustering performance is evaluated via the following criteria, *F*_1_, *Precision*, and *Recall*. The visualized results provide a comparison between the denoised images and ground truth structure, which can be observed directly.

### 4.3. Denoising Performance

We first compare the denoising performance of the new model with the state-of-the-art denoising methods in terms of PSNR (peak signal-to-noise ratio), a common criterion for measuring the denoising quality. The higher the PSNR, the better the quality of the reconstructed image. In this experiment, we use the simulated images of 5wth, 5gjq, and 5k0y (SNR = 0.1) as the test datasets, and compare the PSNR scores obtained by CDAE and the following 7 methods:

- Filter-based denoising, including NLFMT (Kumar, 2013), BM3D (Dabov et al., 2008) and PID (Knaus and Zwicker, 2014);
- Sparse coding-based denoising, NCSR (Dong et al., 2013);
- Effective priori-based method, PCLR (Xu et al., 2017)
- Deep learning-based method, DnCNN (Zhang et al., 2017) and a single denoising autoencoder, namely single DAE, which has the same model architecture as the autoencoder used in each block of CDAE.

The results are listed in Table 1, and the denoised images are shown in Supplementary Table 1. We use histogram equalization enhancement (HEE) in our method because the output gray values concentrate in a narrow range and the output is sparse. Specifically, the gray values of our model outputs concentrate in a narrow range, and HE can help remap the gray values to a wider range. HEE is commonly used in signal processing and does not modify the main property and features of denoised images. In order to examine the effect of histogram equalization enhancement, we consider two versions of the 6 existing methods, i.e., with and without HEE. Among the 8 methods, CDAE achieves the highest PSNR on 5wth, which is the hardest one among the three proteins, because protein 5wth is small and it has no distinct structural characteristics (as can be seen in Figure 3). For 5k0y, CDAE performs very close to the best method, NLFMT (8.2143 vs. 8.2640); and for 5gjq, CDAE ranks the third place. The histogram equalization enhanced NLFMT achieves the best results on both 5k0y and 5gjq. However, its HEE version performs not stable, as the PSNR values decreases dramatically on 5wth. For most of the methods, HEE leads to reduced PSNRs. Overall, CDEA is a very competative method compared with the existing image denoising methods. Also, through the denoised images, we find that CDAE gets more sparse images than others, thus the specific structural features will be enhanced. Interestingly, our cascade model outperforms the single denoising autoencoder on all the three data sets, indicating that reducing noise progressively would be a practical strategy for handling very-low-SNR images.

**Table 1:** Denoising result comparison for eight methods.

**Method**	**5k0y**	**5wth**	**5gjq**
PCLR	7.22/8.18	6.78/5.66	6.85/8.21
PID	6.87/5.33	6.48/4.95	6.50/5.22
NLFMT	7.15/**8.26**	6.89/5.64	6.74/**8.55**
BM3D	7.05/5.13	6.66/5.22	6.56/6.87
NCSR	7.09/5.37	6.58/4.92	6.55/5.29
DnCNN	7.02/5.38	6.58/4.99	6.52/5.32
Single DAE	7.55	6.77	6.88
CDAE	8.21	**6.92**	7.07

*The numbers before and after “/” denote the PSNR values without and with histogram equalization enhancement (HEE). Both single DAE and CDAE include the HEE step, thus their PSNRs are obtained after HEE. The best values are in bold*.

### 4.4. Clustering Performance

Since our ultimate goal is to improve the clustering performance, so as to get better mean images for 3D structure reconstruction, we cluster the denoised images with some conventional unsupervised algorithms, i.e., *k*means and hierarchical clustering (HC), and compare the accuracy with 6 other methods, which fall into two categories:

1. Traditional methods: *k*means (working on original images), HC (working on original images), PCA+*k*means (working on principle components of the original images) and CL2D (implemented in XMIPP);
2. Deep model based methods: CAE+*k*means (convolutional autoencoder with *k*means), AAE+*k*means (adversarial autoencoder with *k*means, the generator of AAE is a convolutional autoencoder, Makhzani et al., 2015), and DAE+*k*means (denoising autoencoder with *k*means). For the first two methods, latent representations extracted from the middle layer of the convolutional autoencoder are used for clustering, and both inputs and outputs are the original test images; while for DAE, the mean image (averaged over 30 nearest neighbors) for each original image serves as target output, and the outputs of decoder are used in clustering (note that it is different from the original denoising autoencoder proposed by Vincent et al. (2008) as there is no clean target for test data).

All the convolutional autoencoders in the compared deep models (CAE, DAE, and the generator of AAE) have almost the same architecture as the single blocks in our model. We use rmsprop optimizer and train the model by 20 epochs, while in AAE we add extra GAN training procedure to set constraints on latent variables. We also use rmsprop optimizer and train the model by 1500 iterations.

Table 2 shows that our model outperforms other methods at all of the three datasets, indicating that deep-models have great potential serving as image denoising tools. The detailed discussions are as follows.

**Table 2:** Clustering result comparison*.

**Method**	**Measure**	**5gjq**	**5wth**	**5k0y**
	*F*_1_	0.76	0.29	0.54
*k*means	Precision	0.68	0.25	0.43
	Recall	0.80	0.34	0.59
	*F*_1_	0.79	0.29	0.56
HC	Precision	0.74	0.27	0.51
	Recall	**0.84**	0.33	0.65
	*F*_1_	0.76	0.29	0.72
PCA+*k*means	Precision	0.67	0.26	0.63
	Recall	0.78	0.34	0.72
	*F*_1_	0.30	0.28	0.29
CL2D	Precision	0.29	0.27	0.27
	Recall	0.34	0.33	0.30
	*F*_1_	0.77	0.3	0.54
CAE+*k*means	Precision	0.7	0.26	0.42
	Recall	0.8	0.39	0.59
	*F*_1_	0.40	0.34	0.59
DAE+*k*means	Precision	0.36	0.32	0.47
	Recall	0.45	0.37	0.75
	*F*_1_	0.4	0.29	0.46
AAE+*k*means	Precision	0.26	0.26	0.41
	Recall	0.32	0.35	0.47
	*F*_1_	**0.81**	0.94	0.75
CDAE+HC	Precision	**0.79**	0.94	0.73
	Recall	0.80	0.93	**0.77**
	*F*_1_	0.76	**0.95**	0.76
CDAE+*k*means	Precision	0.76	**0.95**	**0.75**
	Recall	0.78	**0.95**	0.77

*HC and kmeans denote hierarchical clustering and kmeans method working with the raw images, respectively; PCA+kmeans denotes clustering of principle components via kmeans; CAE and AAE denote the conventional convolutional autoencoder and adversarial autoencoder, respectively; DAE denotes the convolutional autoencoder with the original test images as input and their mean images within the neighborhood as output (no pre-training), and CDAE denotes our model. Bold values means that they are the maximum metrics value in this dataset.

Among the first four traditional methods, PCA obtains the best results on both 5gjq and 5k0y. Although it is a simple linear transformation, PCA captures the key features that are helpful for clustering the images.

The last five methods are all based on autoencoders, while their performance differs a lot. AAE does not perform well in this task, mainly due to the intrinsic difficulties in the training of the model, which restricts its applications. AAE obtains a lower accuracy even than the traditional methods. As the latent feature vector is a compact representation for the image with much lower dimensionality, if the representation is not good, the clustering performance may be even worse than using original images.

According to the accuracy of CAE, the latent representations could also be useful in the clustering of cryo-EM images, but they also try to reconstruct the noisy patterns, thus may not yield a satisfying result.

DAE has much lower accuracy than CDAE, suggesting that the average images of the original images may not be a good choice for the reconstruction target. By contrast, CDAE adopts a two-phase learning strategy and a cascade structure, which both contribute to the good performance.

CDAE+*k*means and CDAE+HC have very close performance, indicating the robustness of the extracted representations. An interesting result is that our model achieves significantly better accuracies on 5wth. We find that this molecule is relatively small compared to two others, and presents as a denser form in the central area of the images, which may increase the difficulty in clustering. Except CDAE, all the other methods almost group the images into one cluster. The results demonstrate that CDAE captures the discriminant features rightly, thus greatly enhances the performance.

### 4.5. Visualized Results

**Figure 4:**
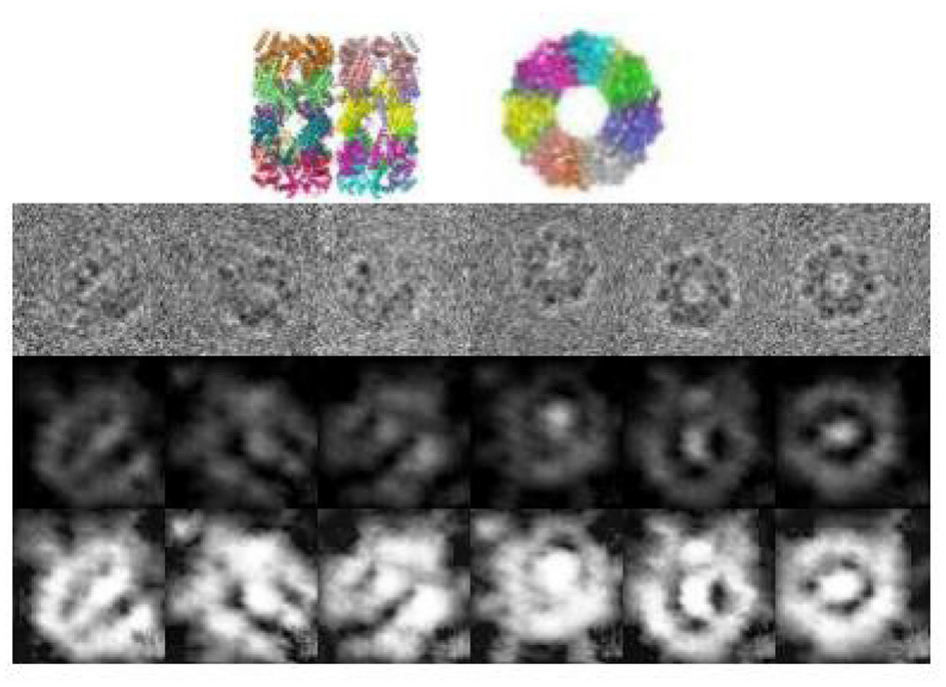
Denoised images of GroEL in different orientations. The 1st row shows the original images, The 2nd row shows the projections of the ground truth structure, the 3rd row shows the denoised images, and the last row shows the denoised images processed by histogram equalization.

As mentioned in section 4.1, we download a data set of protein GroEL from EMDB without corresponding clean images or orientation information. Therefore, clustering or denoising performance can not be evaluated, thus we present the visualized result. Figure 4 shows some examples of the denoised images. It can be observed that the images are consistent with the true structure, and can differentiate between the projection angles.

## 5. Discussion

The proposed CDAE model involves both pre-training and fine-tuning. Benefitting from the abundance of 3D structure simulation software, it is convenient to generate projection images from pre-defined orientations for a certain biomolecule. Therefore, the simulated cryo-EM images could serve as a kind of supervision in the learning algorithms. Furthermore, the mean images can be used for fine-tuning, because the averaging operation can effectively reduce random noisy, and many cryo-EM data processing algorithms use it to enhance the image features, like EMAN2 (Tang et al., 2016). We also design the denoising model in a cascade structure based on the following concern. The cryo-EM images often have a high noise ratio. During the pre-training phase, if we choose a low SNR for the simulated data, apparently the input and target output differ a lot, and it is hard for the layers to adapt the noise; but if we set a high SNR, although the deep network could easily learn the noisy pattern, it does not accord with the real case, and the quality of learning would be affected. Therefore, we want to reduce noisy in a progressive manner and design a cascade of denoising autoencoders to reduce the noise step by step.

The quantitative and visualized experimental results in the previous sections demonstrate the good performance of CDAE, which is attributed to the advantages on model design. Comparing with the DnCNN model, our model has a deeper network architecture, which may have greater capacity on feature representation; and comparing with the single DAE model, our model benefits from the cascade design, which can gradually and smoothly guide the denoising process, thus making the denoising process more controllable and leading to better denoising effect.

The proposed model is closely related with denoising autoencoder (DAE) (Vincent et al., 2008) and Stacked Denoising Autoencoders (SdA) (Vincent et al., 2010). Actually, the components of our model, the autoencoder in each block, has the same architecture of DAE, and both of them are fed with corrupted images and rendered to reconstruct clean images. However, the objectives of these two methods are fundamentally different. Unlike our model, DAE aims to learn robust features, and use the pre-trained autoencoder as an initialization for subsequence supervised learning tasks. Therefore, the DAE model is fine-tuned by training data in a supervised manner, while our model is fine-tuned in a pseudo-supervised manner, in which the mean images are assumed to be the reconstruction targets.

Besides, our model also looks similar with the SdA model (Vincent et al., 2010). However, the architecture of these two models are very different. Our model consists of three blocks, each block has the same component autoencoder. And for Blocks 2 and 3, they are red by the outputs (denoised images) from previous blocks; while in SdA, it is the latent representation rather than the output being passed to the next autoencoder. And, SdA has the same object of DAE and also receives a supervised fine-tuning.

Although CDAE achieves a good performance on PSNR metric and visual results, there is still a big gap between the denoised images and the ground truth clean images. There are two possible reasons. First, the added noise in simulated data may be very different from true noise. The noise in real cryo-EM images usually has complex sources, while the simulated images are added with Gaussian noise or noise with single types of distribution. Second, the neighboring images that are used for computing mean images may be selected inaccurately, as the images are extremely noisy and it is difficult to measure image similarity. Therefore, our future work will focus on the generation of noisy images to improve the pre-training process and investigate the similarity metric of images.

## 6. Conclusion

In this study, we propose a cascade of denoising autoencoders to reduce noise in cryo-EM images and enhance the clustering performance. This model contains 3 denoising blocks, and each block contains a denoising autoencoder. The 3 blocks learn simulated images from low SNR to medium SNR, medium SNR to high SNR, high SNR to clean data, respectively. After the pre-training, each autoencoder is fine-tuned by using the mean images. We provide both quantitative and visualized results on both simulated and real data sets. In the quantitative experiments, we compare the PSNR values with other denoising algorithms and evaluate the clustering performance, while in visualization evaluation, we compare the denoised images with the ground truth protein structure. The experiments show that our method achieves significant better performance of denoising and clustering than the state-of-the-art methods on the highly noisy cryo-EM images.

## Data Availability Statement

Publicly available datasets were analyzed in this study. This data can be found here: ebi.ac.uk/pdbe/emdb/empiar/entry/10029/.

## Author Contributions

HL and YY designed the model, analyzed the results, and wrote the manuscript. HL conducted the experiments. All authors contributed to the article and approved the submitted version.

## Conflict of Interest

The authors declare that the research was conducted in the absence of any commercial or financial relationships that could be construed as a potential conflict of interest.
