# Supplementary material for: CDAE: A Cascade of Denoising Autoencoders for Noise Reduction in the Clustering of Single-Particle Cryo-EM Images
Source: Front Genet. 2021 Jan 20;11:627746. doi: 10.3389/fgene.2020.627746 (PMC7854571; doi:10.3389/fgene.2020.627746)
Supplement: Supplementary file 1 [file Data_Sheet_1.PDF]

Supplementary Materials

Table S1. Comparison of denoising performance on three test sets.

Dataset / Method	5k0y	5wth	5gjq
Clean image	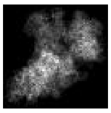	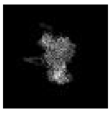	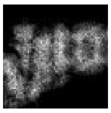
Noisy image	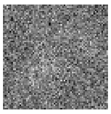	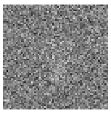	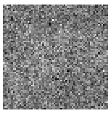
PCLR	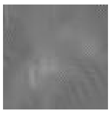	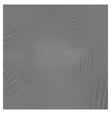	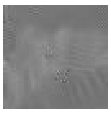
PID	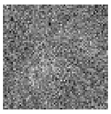	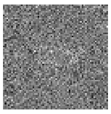	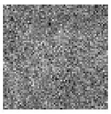
NLM	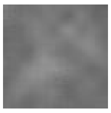	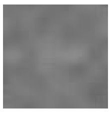	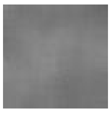
BM3D	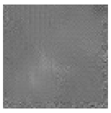	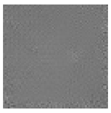	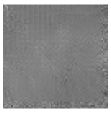
NCSR	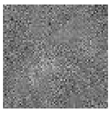	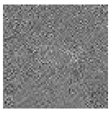	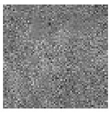
DnCNN	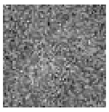	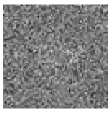	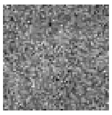
Single DEA	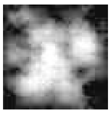	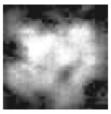	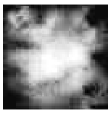
CDAE	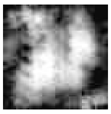	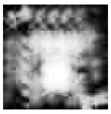	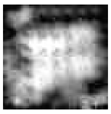

The first two rows show clean images and original noisy images, and the following rows show denoised images by the 8 methods.
